# Characterization of dorsal root ganglion neurons cultured on silicon micro-pillar substrates

**DOI:** 10.1038/srep39560

**Published:** 2016-12-23

**Authors:** Tihana Repić, Katarina Madirazza, Ezgi Bektur, Damir Sapunar

**Affiliations:** 1 Laboratory for Pain Research, School of Medicine, University of Split, Croatia; 2 Speech and Hearing Research Laboratory, School of Medicine, University of Split, Croatia; 3 Histology and Embryology Department, School of Medicine, Eskisehir Osmangazi University, Turkey

## Abstract

Our study focuses on characterization of dorsal root ganglion (DRG) neurons cultured on silicon micro-pillar substrates (MPS) with the ultimate goal of designing micro-electrode arrays (MEAs) for successful electrophysiological recordings of DRG neurons. Adult and neonatal DRG neurons were cultured on MPS and glass coverslips for 7 days *in vitro.* DRG neuronal distribution and morphometric analysis, including neurite alignment and length, was performed on MPS areas with different pillar width and spacing. We showed that MPS provide an environment for growth of adult and neonatal DRG neurons as permissive as control glass surfaces. Neonatal DRG neurons were present on MPS areas with narrow pillar spacing, while adult neurons preferred wider pillar spacing. Compared to the control glass surfaces the neonatal and adult DRG neurons in regions with narrow pillar spacing range developed a smaller number of longer neurites. In the same area, neurites were preferentially oriented along three directional axes at 30°, 90° and 150°. MPS architecture influenced growth directionality of all main DRG neuronal subtypes. We can conclude that specific micro-pillar substrate topography affects the morphology of DRG neurons. This knowledge can enable development of MEAs with precisely defined physical features for various neuroscience applications.

Dorsal root ganglion (DRG) neurons are an important site for pathophysiological changes that lead to neuropathic pain^1^. Following nerve injury or inflammation, DRG neurons may become an important source of nociceptive signaling through increased neuronal excitability and generation of ectopic discharges^2^,^3^.

Beside the classical electrophysiological techniques the activity of injured DRG neurons can be recorded by using micro-electrode arrays (MEAs), a technology that has been used by an increasing number of neuroscience laboratories^4^,^5^,^6^. With advanced MEAs based on integrated complementary metal oxide semiconductor (CMOS) any neuron grown over custom arrays can be recorded at high spatio-temporal resolution allowing us to better understand some aspects of its altered electrophysiological properties^7^. An important feature of this methodology is the ability to record, for relatively long periods of time, large populations of neurons and their neurites on several tens of microelectrodes arranged on the culture substrate^8^.

In order to use MEAs for a wide range of neuroscience applications, including neuro-regenerative medicine, construction of neural networks and electrophysiology studies, it is crucial to design and produce micro-pillar substrates (MPS) with specific topography that will provide favorable growth and desirable morphology of cultured neurons or precise guidance and positioning of their neurites^9^,^10^,^11^,^12^. In recent years neural response to various types of topography of the cell adhesion substrata was intensively studied. For this purpose, numerous isotropic and anisotropic, micro- and nano-patterned substrates of different rigidity^13^ and geometrical features such as shape (e.g. vertical pili, grooves-lines), size and spatial density^14^,^15^,^16^,^17^,^18^ were produced and used in studies of cellular behavior. The knowledge gained by observing the interaction of neurons with appropriate substrates may allow us to predict or even control their behavior through the fabrication of substrates with precisely defined physical characteristics.

The main aim of this *in vitro* study was to investigate the response of DRG neurons to the silicon MPS isotropic topography consisting of individual areas with pillar structures of different dimensions and density. We focused on the influence of MPS isotropic features on presence and morphology, i.e. neurite alignment, length and branching of the different DRG neuronal subtypes. In addition, both neonatal and adult-derived DRG cells were examined to determine whether sensitivity to topography was age-dependent.

## Materials and Methods

### Micro-pillar substrate (MPS) layout

**Figure 1:**
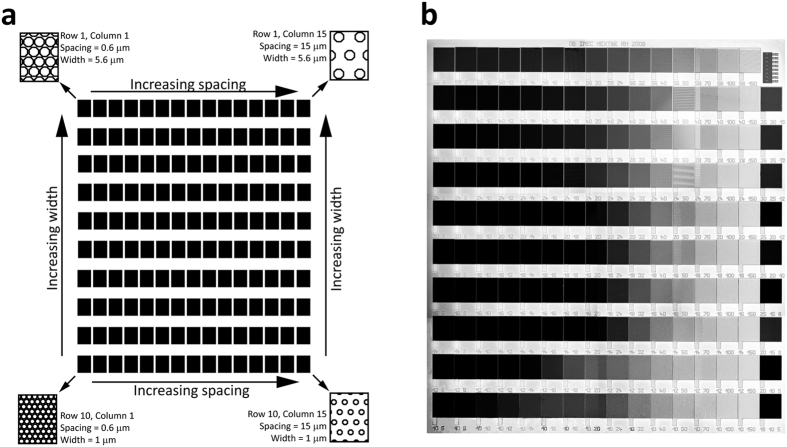
(**a**) Schematic drawing of the substrate layout with 150 areas with 3 μm high hexagonal pillar structures of different width and spacing. The pillar width ranged from 1–5.6 μm in the vertical direction, while the spacing ranged from 0.6–15 μm in the horizontal direction. The areas with pili are separated with a flat border area. (**b**) Microphotograph of the substrate.

The substrates used for the cell cultures were fabricated from silicon wafers according to a previously described procedure^19^. The 8 × 8 mm substrate consists of 150 individual areas micro-patterned with hexagonal pili of different width and spacing. The pillar width range is from 1–5.6 μm (1, 1.2, 1.4, 1.6, 1.8, 2, 2.4, 2.8, 4, 5.6 μm) in the vertical direction, while the pillar spacing is from 0.6–15 μm (0.6, 0.8, 1, 1.2, 1.4, 1.6, 1.8, 2.0, 2.4, 3.2, 4, 5, 7, 10, 15 μm) in the horizontal direction, as shown in Fig. 1. The pillar height was kept constant at 3 μm.

### Dorsal root ganglia (DRG) neurons isolation, dissociation and cultivation

DRG neurons were isolated from adult male (170–220 g) and neonatal 5 to 7 day-old Sprague-Dawley rats. Each DRG neuronal culture was obtained from only one rat. Rats were purchased from University of Split Animal Facility where they were raised in a controlled environment (food and water *ad libitum*, temperature 22 ± 1 °C, light schedule: 12 h of light and 12 h of dark) and housed in pairs in plastic cages with sawdust and corn bedding. All animal experiments and protocols were approved by the University of Split Animal Care and Use Committee, Veterinary and Food Safety Office of the Ministry of Agriculture (permit number HR-POK-022) and followed the International Association for the Study of Pain (IASP) ethical guidelines.

DRG neurons from adult rats were harvested during inhalation anesthesia with 2% isoflurane in oxygen (Forane, Abbott Laboratories Ltd., Queenborough, UK). The spine was exposed after midline skin incision and two lateral incisions through muscles. Muscle tissue was removed and spinal column opened until ganglia were clearly visible. The left and right L4 and L5 DRGs were dissected out and placed into the 35 mm sterile Petri dish containing ice cold Hank’s Balanced Salt Solution (HBSS, calcium/magnesium-free, Invitrogen, Carlsbad, CA, USA). The remaining nerves and connective tissue were carefully removed under the stereomicroscope.

Neonatal rats were euthanized by decapitation following cold anesthesia. The spinal column was removed from the rat and transferred to a small Petri dish containing cold HBSS. After cleaning the remaining tissue, the spinal column was opened by cutting through the dorsal spinal vertebrae to expose the spinal cord. Each DRG was isolated from the spinal cord by peeling it away from the membranes and cutting of the roots as close to the ganglion as possible.

Dissociation procedure was identical for adult and neonatal DRGs. It was performed by incubating DRGs in Dulbecco’s modified Eagle medium (DMEM, Invitrogen, Carlsbad, CA, USA) containing 0.07% trypsin (Sigma-Aldrich, St. Louis, MO, USA), 0.01% Liberase™ (Roche, Basel, Switzerland) and 38 U/ml DNase (Roche, Basel, Switzerland) for 60 minutes at 37 °C in shaking water bath. Digestion was terminated by adding 0.25% trypsin inhibitor (Roche, Basel, Switzerland). Ganglia were triturated with a fire-polished Pasteur pipette until the suspension was homogenous. The cell suspension was then centrifuged for 6 min at 6000 rpm and DRG pellet was resuspended in Neurobasal-A medium supplemented with 2% B27 supplement, 0.5 mM L-Glutamine, 0.02 mg/ml gentamicin (Invitrogen, Carlsbad, CA, USA) and 100 ng/ml nerve growth factor 7 S (Corning, NY, USA).

The DRG neurons were plated on glass coverslips or micro-pillar substrates (MPSs). The 13 mm glass coverslips (Thermo Fisher Scientific, Boston, MA, USA) were sterilized by autoclaving for 20 min at 120 °C, while silicon MPSs were cleaned with acetone overnight, rinsed with 70% ethanol and left to dry under sterile conditions. Both cultivation substrates were then placed in 24-well plates (TPP, Trasadingen, Switzerland) and coated with 0.2 mg/ml poly-L-lysine (WT 70,000–150,000, Sigma-Aldrich, St. Louis, MO, USA) at room temperature overnight. Prior to cell seeding, substrates were cleaned with sterile water and air-dried in a tissue culture hood. Cells were counted using a Bürker-Türk chamber and 100 μl of DRG neuron suspension (≈5000–15000 cells) were seeded on each poly-L-lysine-coated coverslip or MPSs placed in well. Plates were then placed in incubator for 10–15 min (37 °C, 5% CO_2_) after which the rest (1 ml) of the warm (37 °C) growth medium was added. During the 7-day cultivation process, half of the medium was changed every 2–3 days.

### Immunofluorescence and scanning electron microscopy (SEM)

After the 1^st^, 3^rd^ and 7^th^ day *in vitro* (further in text: 1DIV, 3DIV, 7DIV), DRG cultures were fixed with 4% paraformaldehyde in 0.01 M phosphate buffered saline (PBS, pH = 7.4) for 20 min. The cells were washed three times with PBS, permeabilized with 0.1% Triton-X100 (Millipore, Billerica, MA, USA) in PBS and incubated in blocking medium [4% goat or donkey serum (Dako, Glostrup, Denmark) in 0.1% Triton-X100 PBS] for 60 min at room temperature. Each of the subsequent wash and incubation steps were performed in 0.1% Triton-X100 PBS buffer.

Specific DRG neuronal subtypes were identified by simultaneous staining of NeuN and N52 or IB4 or CGRP (details on primary antibodies are listed in Table 1).

The cells were incubated overnight at 4 °C in dark humidity chamber, with a mixture of antibodies, or in the case of IB4 with the mixture of primary antibody and IB4 conjugate. The next day, cells were incubated for 60 min at room temperature with corresponding secondary antibodies and conjugates (Table 1). Nuclear staining was performed using 5 μg/ml DAPI (Invitrogen, Carlsbad, CA, USA) for 2 min at room temperature in the dark. After final washing in PBS, slides were mounted with Shandon Immu-Mount (Thermo Fisher Scientific, Boston, MA, USA) and visualized under Olympus BX51 fluorescence microscope with appropriate filters for selectively detecting green and red fluorescence using an Olympus DP71 digital camera.

For SEM imaging, samples were fixed in 4% paraformaldehyde for 20 min, rinsed three times with PBS, and three times for 5 min with distilled water. Subsequently, they were dehydrated with a graded ethanol series of the following dilutions in dH_2_O: 25% (1 × 5 min) − 50% (1 × 10 min) − 75% (1 × 10 min) − 95% (1 × 10 min) − 100% (3 × 10 min), and left to dry under the air flow. Samples were observed using Jeol JSM-5200 (Tokyo, Japan) scanning electron microscope.

### Analysis of DRG neuronal distribution on MPS

Distribution of the NeuN positive neurons was determined after three days in culture for each area of MPS and expressed as percentage of total number of neurons on all pillar areas of specific MPS. The results were averaged for five neonatal and five adult cultures and plotted as a function of pillar width and spacing using SigmaPlot software (Systat Software, San Jose, USA) to get preferential distribution of the DRG neurons across MPS surface. According to micro-pillar spacing, MPS was divided into three vertical bins (0.6–1.4 μm; 1.6–3.2 μm; 4–15.0 μm) and according to pillar width in three horizontal bins (1–1.4 μm; 1.6–2.4 μm; 2.8–5.6 μm). The density of plated DRG neurons was calculated as a relative number of cells per mm^2^ and compared between vertical and horizontal MPS bins and control glass surfaces. Values were normalized by the surface area (0.2 mm^2^ for each of 150 MPS areas and 132.7 mm^2^ for the control glass).

### Measurement of neurite number and length

Unlike other cells, neurons have a large network of axons and dendrites, which are referred non-specifically as neurites when grown in culture. The “NeuronJ” plug-in (version 1.4.3) of NIH-ImageJ software (version 1.50)^24^ was used to quantify the average neurite length of each neuron at different time points and different MPS bins. Neurites grown on glass coverslips were used as a control. Briefly, 8-bit grayscale images of fluorescent neurons with identifiable neurites were loaded into the software and calibrated according to the image magnification. Average length of the neurites was obtained by manually tracing the length of all neurite outgrowths from neuron’s cell body, divided by total number of neurites per neuron. Neurite lengths and total number of neurites were averaged across all neurons in each MPS bin or glass coverslip and plotted.

### Quantification of neurite alignment using Fourier image analysis

To determine the influence of pillar dimensions on neurite alignment of the cultured DRG neuronal cells, images of immunostained neurons were analyzed using the Fast Fourier Transform (FFT) algorithm. The FFT function converts directional spatial information present in an original image into a mathematically defined frequency domain. The resulting FFT output image maps the rate at which pixel intensities change across the original data image, giving us the information about the orientation of objects present in the original data image^25^. In other words, an image with no alignment will show constant pixel intensity absent of peaks, indicating no specific directionality. Conversely, an image containing structures aligned in one direction will have higher pixel intensities along that direction, which would be plotted as a peak in that direction^26^,^27^. In our experiment, quantification of neurite alignment on MPS grouped into 3 horizontal and vertical bins and control glass coverslips, was accomplished by performing FFT analysis using NIH-ImageJ software supported by the “Oval Profile” plug-in (authored by Bill O’Connell, https://imagej.nih.gov/ij/plugins/oval-profile.html). For specific areas of each bin, three digital images of immunostained DRG neurite outgrowths were taken. Images were converted to 8-bit grayscale TIFF files using Adobe Photoshop CS3 software (Adobe Photoshop CS3, Adobe Systems Incorporated, San Jose, USA) and cropped to 512 × 512 pixels. As FFT always treats an image as if it is part of a periodically replicated array of identical images extending to infinity, strong edge effects will appear and add spurious information to the peaks of an alignment plot^25^. To eliminate those effects, a radial feather mask was applied onto each image by using the elliptical marquee tool and setting the feather options to 20 pixels and 100% density. Prepared images were then processed with the FFT function of the ImageJ software to generate the FFT image. The applied ring analysis of “Oval Profile” plug-in extracts data from the frequency domain of the FFT image and provides radial sum intensity values for all 360 angles. Since FFT is symmetrical about the horizontal axis, pixel intensities were summed along each degree and plotted between 0° and 180°^28^.

### Statistical analysis

Data were analyzed with GraphPad Prism 5 software (GraphPad Software, Inc., La Jolla, CA, USA). Differences in distribution were tested using t-test or one-way ANOVA followed by a Bonferroni *post-hoc* test. A p value less than 0.05 was considered significant.

## Results

### MPS is a favorable surface for the growth of DRG neurons

DRG cells from neonatal and adult rats were cultured on MPS with different micro-pillar configurations and compared to control neurons cultured on glass coverslips. The cultured DRG neurons adhered firmly to the chip surface and did not easily detach during medium change and staining procedures. The overall growth characteristics of DRG neurons on MPS were similar to their growth pattern on conventional substrates, i.e. borosilicate glass coverslips.

**Figure 2:**
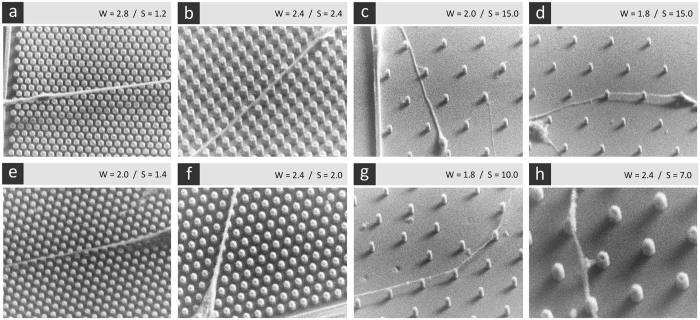
Representative scanning electron microscopy (SEM) images of (**a–d**) neonatal and (**e–h**) adult DRG neurons showing the interaction between micro-fabricated pillar structures and neurites after 3DIV. Examples of neurite-pilus interaction on MPS area with: (**a,e**) narrow inter-pillar spacing (0.6–1.4 μm), (**b,f**) midrange pillar spacing (1.6–3.2 μm), and (**c,d,g,h**) wide pillar spacing (4–15 μm). Scale bar: 10 μm. Legend: W - width; S - spacing.

To determine whether the neurites are growing between the pillar structures or on the top of them in specific areas of MPS, scanning electron microscopy images of DRG neurons were obtained. Figure 2 is showing the interaction of neurites with pillar structures in specific MPS areas. In the regions with narrow pillar spacing (Fig. 2a,b,e,f), neurites were growing directly on pillar tips. As the inter-pillar spacing increases, neurites started to invade and grow on the flat surface between the pili. However, the contact with pili was not completely lost. Some neurites touched the bottom of pili and showed the tendency to “climb” again on the top of them (Fig. 2c,d,g,h). This partial loss of contact guidance in regions with larger inter-pillar spacing leads to reduction of neurite alignment in this particular area of pillar array (described later in section about neurite outgrowth directionality). The observed behavior was similar for neonatal and adult neurons.

### MPS architecture influences the distribution of DRG neurons in culture

**Figure 3:**
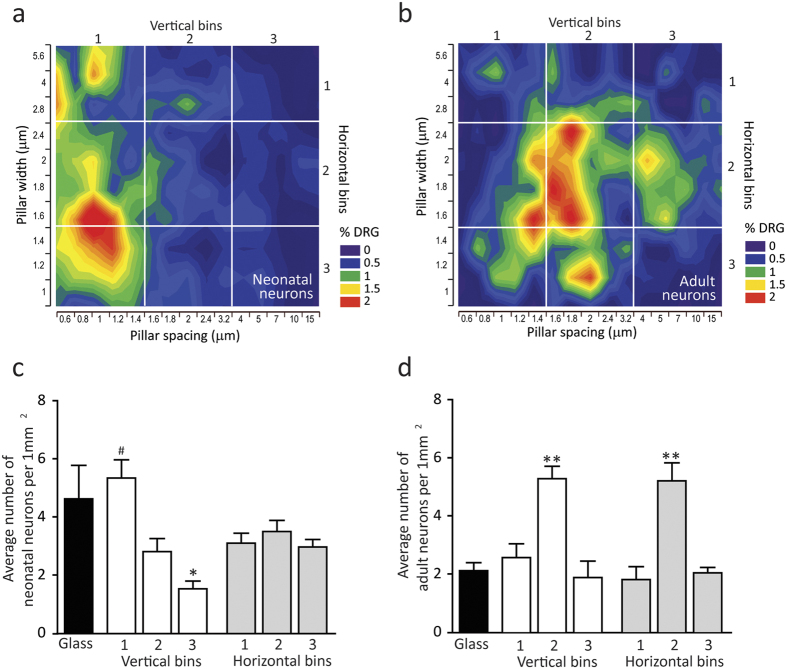
Effect of MPS architecture on distribution of DRG neurons. The surface plots of (**a**) neonatal and (**b**) adult DRG neurons depict neuronal distribution as a function of pillar width and spacing. The white lines mark the division of the MPS in three horizontal and three vertical bins. The average number of (**c**) neonatal and (**d**) adult DRG neurons per one square millimeter in specific bins shows preferential distribution in the area with smaller pili and their narrow spacing for neonatal neurons and in the central area for adult neurons. Data are means ± SEM. Legend: ^#^indicates significant difference between first vertical bin and two remaining vertical bins; *difference between glass and third vertical bin; **difference between glass and second vertical and horizontal bin.

In order to investigate the influence of substrate architecture on preferential growth of DRG neurons, the percentage of NeuN positive neurons located on each of 150 individual MPS areas was calculated from 5 independent experimental cultures with adult (n = 1011 neurons) and 5 experimental cultures with neonatal neurons (n = 1966 neurons). The surface plot of DRG neurons, both neonatal (Fig. 3a) and adult (Fig. 3b), revealed a preferential distribution in regions with specific pillar geometries. The distribution of neurons is presented only for 3DIV because the same growth pattern was observed after 1DIV and 7DIV.

The average density of neonatal DRG neurons per square millimeter was significantly higher than density of adult neurons (4.6 vs. 2.1 per 1 mm^2^ respectively; t-test, P < 0.001). The affinity for the regions with small pillar width and spacing was more pronounced in neonatal DRG neurons. The neuronal density in that region was higher than in remaining parts of the MPS (Fig. 3c). However, adult neurons were mostly located in the central region of the MPS. The average density per square millimeter in that region was significantly higher than density on glass coverslips and other regions of the MPS (Fig. 3d).

### MPS architecture elicits specific directional DRG neurite growth

**Figure 4:**
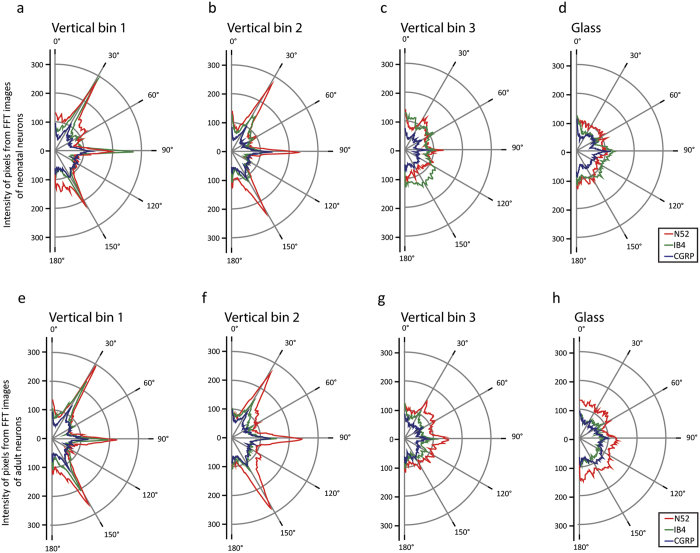
Effect of pillar spacing on neurite alignment in different subtypes of: (**a–d**) neonatal and (**e–h**) adult DRG neurons. The radial values represent the fluorescence intensity of pixels from FFT images along the same angle. Angles are from 0°–180°. A total number of 360 neurons were analyzed.

In order to describe whether MPS topography has an effect on neurite outgrowth directionality, DRG neurons were stained with N52, CGRP and IB4 as main markers of different DRG neuronal subtypes. Analysis showed that specific inter-pillar spacing had a strong influence on neurite outgrowth directionality (Fig. 4), while pillar width did not show any effect on DRG neurite orientation (data not shown). The strong influence was observed in 1^st^ and 2^nd^ vertical bins with pillar spacing between 0.6–1.4 μm (Fig. 4a and e) and 1.6–3.2 μm (Fig. 4b and f). The most common neurite orientation was along three axes at 30, 90 and 150 degrees. This alignment was observed in all subtypes of adult and neonatal DRG neurons. Conversely, the pillar architecture of the 3^rd^ bin with pillar spacing between 4–15 μm (Fig. 4c and g), did not induce preferential neurite growth in any direction and was similar to the growth observed on glass coverslips. On control glass surfaces DRG neurons showed an extensive, multidirectional neurite outgrowth with randomly oriented neurites (Fig. 4d and h).

**Figure 5:**
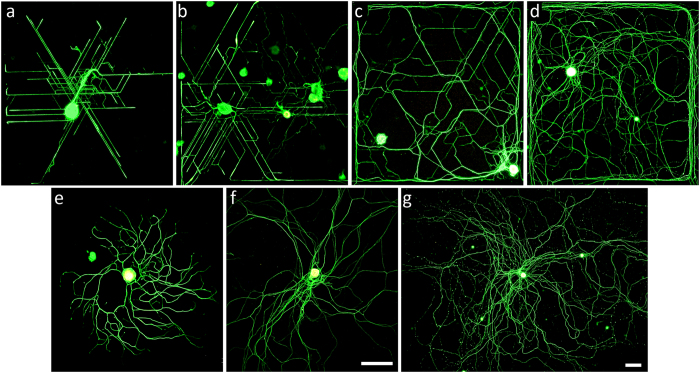
Effect of surface topography on neurite alignment of cultured DRG neurons grown on glass coverslips and different MPS bins. Immunofluorescent images show examples of: (**a**) adult and (**b**) neonatal neurons grown in 1^st^ vertical bin after 1DIV, (**c**) adult neurons grown in 2^nd^ vertical bin after 3DIV, (**d**) adult neurons grown in 3^rd^ vertical bin after 7DIV, and adult neurons grown on control glass surface after (**e**) 1DIV, (**f**) 3DIV and (**g**) 7DIV. Scale bar: (**a–f**) 200 μm and (**g**) 100 μm.

Examples of immunostained neonatal and adult DRG neurons in Fig. 5 show the existence of topographical guidance in 1^st^ and 2^nd^ MPS bins (Fig. 5a–c) and its decrease towards 3^rd^ vertical bin (Fig. 5d). It is clearly visible that control glass surfaces have no topographical guidance capability (Fig. 5e–g). The existence of specific neurite alignment in regions with narrow pillar spacing (1^st^ and 2^nd^ MPS bins) can be partly explained by the availability and closeness of pillar structures as confirmed by analysis of the SEM photos (Fig. 2).

### The spatial density of the pillar structures affects the length and number of neurites

To investigate whether width and spacing of substrate pili have an effect on DRG neurite number and length, a total number of 331 DRG neurons were analyzed. The neurite length was measured after 1DIV and 3DIV, since measurement at 7DIV was impossible due to very long and intertwined neurites extending outside of the field of view.

**Figure 6:**
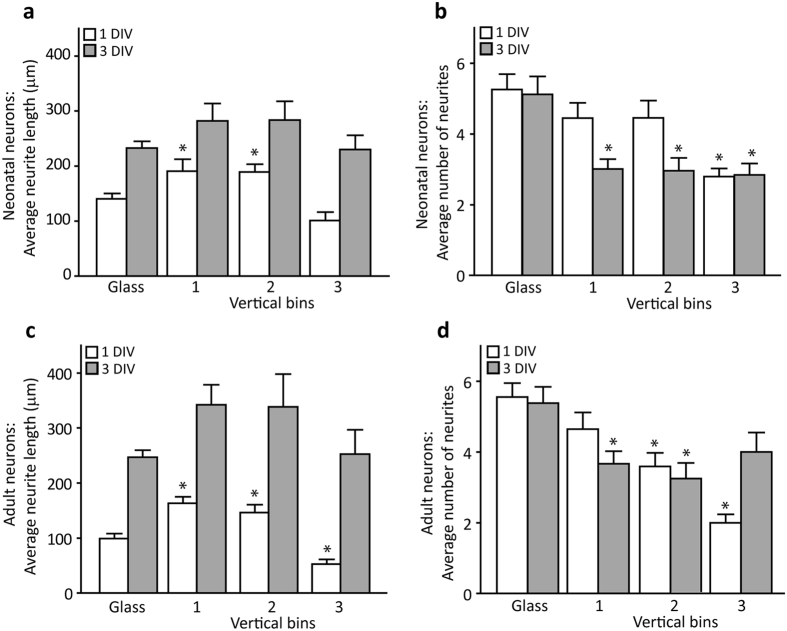
Effect of pillar spacing on (**a,c**) average neurite length and (**b,d**) neurite number after 1DIV and 3DIV in (**a,b**) neonatal and (**c,d**) adult DRG neurons (n = 331). Significant differences between glass and MPS data are indicated by asterisk.

The number and length of the DRG neurites was significantly affected by the spacing between pili but not by pillar width. After 1DIV, neurites were significantly longer in the 1^st^ vertical bin (MPS spacing area of 0.6–1.4 μm) and 2^nd^ vertical bin (MPS spacing area of 1.6–3.2 μm) compared to the control glass surface, for both neonatal (One way ANOVA, main effect F(3,76) = 6.725; P = 0.0004) and adult (One way ANOVA, main effect F(3,84) = 15.32; P < 0.0001) DRG neurons (Fig. 6a and c). In adult DRG neurons the neurite length in the 3^rd^ vertical bin (MPS spacing area of 4–15.0 μm) was significantly shorter compared to neurons grown on glass coverslips. As expected, after 3DIV, all neurites were longer than at 1DIV, but the increase was larger for adult neurons. However, the difference in neurite lengths between neurons grown on glass and on MPS was lost.

The number of neurites per neuron was significantly lower after 1DIV in neonatal DRG neurons located in the 3^rd^ vertical bin compared to other vertical bins and control coverslips (One way ANOVA, main effect F(3,76) = 2.846; P = 0.0431). After 3DIV, the number of neurites decreased in all vertical bins compared to those grown on glass coverslips (One way ANOVA, main effect F(3,70) = 3.090; P = 0.0326) (Fig. 6b). A similar pattern was observed in adult neurons. On 1DIV, the number of neurites per neuron in the 2^nd^ and 3^rd^ vertical bins was lower than those grown on coverslips, as well as in the 1^st^ and 2^nd^ vertical bins at 3DIV in culture (One way ANOVA, main effect F(3,84) = 9.080; P < 0.0001) (Fig. 6d).

These results suggest that MPS provides favorable environment for the growth of neonatal and adult neurons with lower number of longer neurites, especially in areas with pillar spacing between 0.6 and 3.2 μm.

## Discussion

Our results demonstrate that MPS can successfully support normal growth of neonatal and adult DRG neurons. However, preferential growth in specific regions of the MPS was different in neonatal and adult neurons. The neonatal neurons prefer areas with narrow pillar spacing (from 0.6 to 1.4 μm; 1^st^ vertical bin) while adult neurons favor central parts of MPS with wider pili (from 1.6 to 2.4 μm; 2^nd^ horizontal bin) and wider spacing between them (from 1.6 to 3.2 μm; 2^nd^ vertical bin). We do not know if the preferential growth is a result of differential attachment or later survival selectivity.

SEM images revealed that neurites grow by connecting pillar tips in the regions with narrow inter-pillar spacing (vertical bins 1 and 2; from 0.6 to 3.2 μm). This is most likely the cause of specific neurite alignment in those MPS areas. Also, the DRG neurons in these regions developed a smaller number of longer neurites. However, as the spacing between pillar structures increased above 3.2 μm, the effect of topographical guidance diminished as neurites started to partially grow between the pillar structures. Similar results were obtained in other studies with different types of neurons^14^,^16^,^17^,^19^. Only a few studies have examined the impact of the substrate architecture on the growth of DRG neurons and directionality of its neurites. These studies demonstrated that anisotropic substrates like micro-grooved substrates^29^, rows of nanowires^30^, nano-patterned grooves^31^,^32^ or aligned electrospun nanofibers^33^ can direct DRG neurite growth in a specific fashion. In majority of these studies, the anisotropic patterning induced parallel alignment of neurites^29^. Similar results were observed for other types of neurons grown on surfaces with geometrical features. These studies have also shown that periodic geometrical features on surfaces increase total axonal outgrowth and tend to bias growth along certain preferred directions^34^.

To our knowledge, this is the first study that examined the effect of isotropic topography on growth and morphology of neonatal and adult DRG neurons *in vitro*. On isotropic structures, neurites follow existing contacts (pili, pits), but if a new contact is nearby they extend to this contact resulting in a highly aligned distribution of extending neurites^14^,^17^,^35^,^36^,^37^. In our case, neurite alignment along 30°, 90° and 150° radial angles was only obvious in the first two bins. Although others found that neurite growth pattern depends on the cell type^37^,^38^,^39^,^40^ in our study, a similar type of neurite growth was present in all three main subtypes of the DRG neurons, both neonatal and adult.

The most common morphological neuronal changes on patterned substrates include increased neurite alignment^41^,^42^, decreased neurite branching^41^,^42^, increased neurite length^42^,^43^,^44^, and increased polarization^45^,^46^. The extent of these changes depends on the type of neurons as well as the distribution, geometry and physical properties of the substrate.

Because of the many differences between neonatal and adult neurons^47^, we could also expect many differences between their responses to the topography of MPS. For example, the low expression of the integrin in adult neurons^48^ can be the source of differences compared to neonatal neurons. However, our data shows that these differences are minor. In our study, neonatal and adult DRG neurons exhibited enhanced growth onto pillar array with narrow pillar spacing (1^st^ and 2^nd^ vertical bins). The third vertical bin did not have any influence on the length of neonatal neurons but adult neurons from that bin produced a significantly smaller number of neurites compared to neurons grown on glass coverslips. While the length of neurites increased, the number of neurites decreased in all bins for both, neonatal and adult neurons indicating adequate growth potential of DRG neurons. Similar responses of adult and neonatal neurons have been observed when grown on isotropic and anisotropic substrates^32^.

The comparison of our results obtained for DRG cells with results of other studies is difficult because of different MPS material, size, shape and uniformity and also the different technique used for alignment and neurite behavior measurements. However, the results are similar and a tentative trend can be drawn for an increased neurite length and decreased number of neurites for neurons grown on MPS^14^,^19^,^49^,^50^,^51^. We can speculate that the pillar structures act as “anchoring points” for the growth cone, allowing it to make more rapid progress by reducing the frequency of local searches via protrusion-retraction events.

Considerable efforts have been made to investigate how topography affects function and organization of the cells and determine the intracellular mechanism by which neurons translate physical cues to biological response. However, the exact underlying mechanisms are not yet known^36^ and further studies are necessary in this research field. Our results indicate that topographical features can have specific effects on the behavior of DRG neurons. The ability to regulate behavior of the cells interfacing with different *in vitro* substrates in a predictable manner can have a huge effect on tissue engineering, implants, cell-based biosensors, high-throughput microarrays and basic cell biology. For example, Poole *et al*. developed a new method to directly monitor mechanotransduction in primary sensory neurons at defined regions of the cell-substrate interface^52^. Depending on the application, the desired cell function may vary^53^. In our study we focused on patterning ability, which turned out to be similar in neonatal and adult DRG neurons, implying that surface geometry may be a potent cue in directing the regeneration of these neurons. In conclusion, our study demonstrates the feasibility of MPS with specific architecture in supporting DRG growth and control of DRG neurite behavior. This knowledge can enable development of MEAs with precisely defined physical features for successful electrophysiological recordings of DRG neurons.

## Additional Information

**How to cite this article:** Repić, T. *et al*. Characterization of dorsal root ganglion neurons cultured on silicon micro-pillar substrates. *Sci. Rep.* **6**, 39560; doi: 10.1038/srep39560 (2016).

**Publisher's note:** Springer Nature remains neutral with regard to jurisdictional claims in published maps and institutional affiliations.

## Figures and Tables

**Table 1:** Antibodies and conjugates used.

	Target	Manufacturer; Cat. No.; Lot. No.	Dilution used
Primary antibodies			
Rabbit polyclonal anti-neural nuclear antigen (NeuN)	All neurons^20^	Sigma-Aldrich; SAB 4300883; 480132093	1:500
Mouse monoclonal anti-neurofilament 200 kDa (NF200), clone N52	Myelinated A-fiber DRG neurons^21^	Millipore; MAB 5266, 2567008	1:300
Goat polyclonal anti-calcitonin gene-related peptide (CGRP)	Peptidergic A and C-fiber DRG neurons^22^	Santa Cruz Biotechnology; sc-8856; F1714	1:300
Secondary antibody			
Goat anti-rabbit IgG-TR (conjugated to Texas Red)		Santa Cruz Biotechnology; sc-2780; B1914	1:600
Goat anti-mouse IgG-B (conjugated to biotin)		Santa Cruz Biotechnology; sc-2039; B2614	1:600
Donkey anti-goat IgG-FITC (conjugated to fluorescein isothiocyanate)		Santa Cruz Biotechnology; sc-2024; D0114	1:600
Conjugates			
Isolectin B4 (IB4), FITC-conjugated	Nonpeptidergic C-fiber DRG neurons^23^	Sigma-Aldrich; L 2895; 052M4086V	1:100
Streptavidin DyLight 488 conjugate		Abcam; ab134349; GR224458-1	1:600
